# Improvement of Carotenoids’ Production by Increasing the Activity of Beta-Carotene Ketolase with Different Strategies

**DOI:** 10.3390/microorganisms12020377

**Published:** 2024-02-12

**Authors:** Qiaomian Zhou, Danqiong Huang, Haihong Yang, Zeyu Hong, Chaogang Wang

**Affiliations:** 1 Guangdong Technology Research Center for Marine Algal Bioengineering, College of Life Sciences and Oceanography, Shenzhen University, Shenzhen 518060, China; 2100251005@email.szu.edu.cn (Q.Z.); dqhuang@szu.edu.cn (D.H.);; 2 Shenzhen Engineering Laboratory for Marine Algal Biological Development and Application, Shenzhen 518060, China

**Keywords:** β-carotene ketolase, canthaxanthin, fusion tag, molecular chaperone, soluble protein, enzymatic activity

## Abstract

Canthaxanthin is an important antioxidant with wide application prospects, and β-carotene ketolase is the key enzyme involved in the biosynthesis of canthaxanthin. However, the challenge for the soluble expression of β-carotene ketolase is that it hinders the large-scale production of carotenoids such as canthaxanthin and astaxanthin. Hence, this study employed several strategies aiming to improve the soluble expression of β-carotene ketolase and its activity, including selecting optimal expression vectors, screening induction temperatures, adding soluble expression tags, and adding a molecular chaperone. Results showed that all these strategies can improve the soluble expression and activity of β-carotene ketolase in *Escherichia coli*. In particular, the production of soluble β-carotene ketolase was increased 8 times, with a commercial molecular chaperon of pG-KJE8, leading to a 1.16-fold enhancement in the canthaxanthin production from β-carotene. Interestingly, pG-KJE8 could also enhance the soluble expression of β-carotene ketolase derived from eukaryotic microalgae. Further research showed that the production of canthaxanthin and echinenone was significantly improved by as many as 30.77 times when the pG-KJE8 was added, indicating the molecular chaperone performed differently among different β-carotene ketolase. This study not only laid a foundation for further research on the improvement of β-carotene ketolase activity but also provided new ideas for the improvement of carotenoid production.

## 1. Introduction

**Figure 1:**
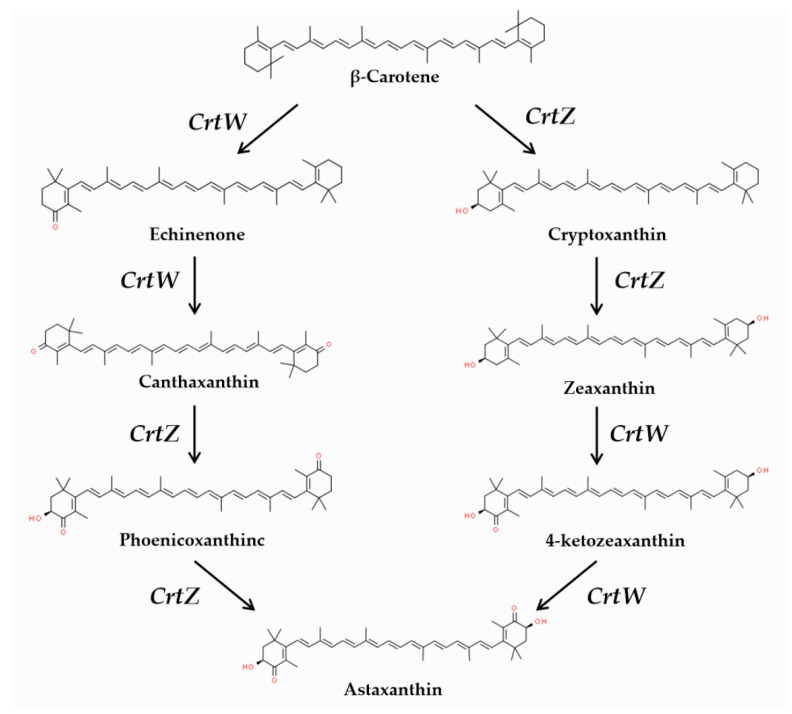
The schematic diagram of astaxanthin biosynthesis from β-carotene. Progressively, the CrtZ gene (β-carotene hydroxylase) adds the hydroxy-group at the end of β-ring, while the CrtW gene (β-carotene ketolase) adds the keto-group at the end of β-ring.

Canthaxanthin is a kind of carotenoid, synthesized by adding two keto-groups to the precursor (β-carotene) using β-carotene ketolase (Figure 1) [1]. Carotenoids are isoprene-like antioxidants acting as pigments in photosynthetic and non-photosynthetic organisms [2] which are essential in biological systems and have a wide range of applications in the fields of environment, food, and medicine [3]. The hydroxylation of carotenoids widely exists in higher plants, but ketonization is limited to a few bacteria, fungi, and some unicellular green algae [4]. Currently, most commercial carotenoids are primarily extracted from various plants and synthetically produced using chemical methods [5]. However, there are some limitations. For example, the large-scale production of natural astaxanthin from microalgae was hindered by the long growth cycle of microalgae and potential contamination [6]. On the other hand, the chemical synthesis of carotenoids generates residual intermediates and by-products that are harmful to human health [7]. Hence, it is necessary to find alternative sources to meet the increasing market demand for carotenoids.

CrtW*_Bsp_* is the β-carotene ketolase derived from *Brevundimonas* sp. strain SD212 (GenBank accession number: BAD99406) with proved function on canthaxanthin biosynthesis from β-carotene [8]. Based on the documentation on Uniprot, there was no signal peptide cleavage site in this protein, but there were four transmembrane regions that are involved in various important biological processes. Since the membrane protein has the characteristics of low abundance and hydrophobicity, it is very difficult to enrich, extract, separate, and identify them [9]. Hence, compared with the analysis of water-soluble proteins, analysis of membrane proteins is more challenging [10]. Gene fusion technology has been able to improve the heterologous expression by overcoming many of these challenges and resulting in soluble active proteins [11]. Subsequently, a series of explorations were also made to improve the soluble production of those membrane proteins, such as the development of a strong promoter [12], co-expression with a molecular chaperone [13], the expression of secretory proteins [14], or fusion proteins [15]. 

Generally, fusion techniques effectively reduce the formation of inclusion bodies in soluble-protein production, thereby enhancing the protein solubility. Furthermore, it also helps to protect recombinant proteins from hydrolysis [16]. The protein fusion tag is typically a peptide sequence with specific functions or properties, which can be connected to the coding sequence of the target protein through genetic engineering methods [16]. Examples of solubility-enhancing tags include Glutathione S-transferase (GST), Small Ubiquitin-like Modifier (SUMO), and Maltose Binding Protein (MBP) [17]. Promoters and other vector elements can significantly affect the intensity and persistence of target gene transcription, and then they can affect the yield of the target protein [18].

In this study, we discussed the effect of different strategies to increase the expression of soluble protein, aiming to improve the activity of β-carotene ketolase in *Escherichia coli* to further develop a more efficient canthaxanthin or even astaxanthin production system. Results in this study can also provide an example for improved production of other member protein in *E. coli*.

## 2. Materials and Methods

### 2.1. Strain, Culture Medium, and Culture Conditions

In this study, *E. coli* strain Top10 (purchased from Shanghai Weidi Biotechnology Co., Ltd., Shanghai, China) was used to construct and proliferate recombinant plasmids, and *E. coli* strain BL21(DE3) (purchased from Shanghai Weidi Biotechnology Co., Ltd.) was used as the host for the production of target proteins and carotenoids. LB medium (10% tryptone, 10% sodium chloride, 5% yeast extract) containing proper antibiotics (100 μg/mL Ampicillin, 100 μg/mL Kanamycin, and/or 34 μg/mL Chloromycetin) was used to culture *E. coli* cells. For cell proliferation, 5 mL cells were grown at 37 °C with 220 rpm in a shaking incubator. For protein induction, 500 mL cells were grown at 15 °C, 30 °C, or 37 °C (depending on the type of plasmids in *E. coli*) with 220 rpm in a shaking incubator.

### 2.2. Construction of Plasmids

Genes encoding β-carotene ketolase used in this study were derived from different species, including *CrtW_Bsp_* from *Brevundimonas* sp. (GenBank accession number: BAD99406), HpW1 and HpW2 from *Haematococcus pluvialis* (GenBank accession number: ADN43073 and GFH09688), CrW1 from *Chlamydomonas reinhardtii* (GenBank accession number: Q4VKB4), and CzW1 from *Chromochloris zofingiensis* (GenBank accession number: AAV41371). Genes were codon-optimized according to the *E. coli* preference and synthesized using GenScript Biotech Co., Ltd. (Nanjing, China). Plasmids were constructed by inserting interested genes into the MCS site of either pET-30a or pCold expression vector using the Gibson Assembly system (Thermo Fisher Scientific Co., Ltd., Massachusetts, USA) or T4 ligase. Plasmids pG∆ZW-*CrtW_Bsp_*/HpW1/HpW2/CrW1/CzW1 and p32*CrtW_Bsp_* were previously constructed in our laboratory. Plasmid pG-KJE8 was purchased from the General Biosystems Co., Ltd. (Chuzhou, China). Detailed information about all plasmids and corresponding primers used for plasmids construction can be found in Supplementary Table S1 and Supplementary Table S2, respectively.

### 2.3. Induced Production of Recombinant Protein

To produce the β-carotene ketolase in *E. coli*, plasmids were transformed into BL21 (DE3) competent cells with or without pG-KJE8, respectively, using a heat-shock method. Positive colonies were selected from the LB plate containing proper antibiotics (100 μg/L Ampicillin, 100 μg/L Kanamycin, and/or 34 μg/L Chloromycetin). Seed cultures were prepared through inoculation of a single colony into 5 mL LB of medium containing suitable antibiotics, and they were grown overnight at 37 °C in a shaker at 220 rpm. The seed culture was then inoculated into 500 mL of LB medium containing 0.5 mg/mL L-Arabinose and 5 ng/mL Tetracycline with the ratio of 1:100. IPTG was added to a final concentration of 0.5 mM when the OD600 reached 0.6. The culture was then maintained in the shaker for an additional 16 h at 15 °C or 37 °C for protein accumulation.

### 2.4. Western Blot Analysis

A Western blot analysis was performed in this study to confirm and compare the protein production in *E. coli*. Firstly, *E. coli* cells were pelleted with centrifugation at 4000 rpm for 10 min at 4 °C. Cells were resuspended with a digestion buffer (containing 0.4 M Tris-HCl pH 8.0, 1 mM dithiothreitol, 5% *w*/*v* glycerol, 1% *w*/*v* protease inhibitor) and disrupted using sonication. The supernatant-containing proteins was collected and filtered through a 0.45 μM membrane into a new 50 mL centrifuge tube, as the protein crude extracts for further analysis. The protein concentration was determined using a BCA Protein Assay Kit (purchased from Beyotime, Shanghai, China). An equal amount of proteins was used for electrophoresis. At the end of electrophoresis, the PVDF membrane was electronically transferred and printed after 30 min. The gel was sealed with milk and incubated with Mouse-anti-His mAb (from purchased from GenScript, Nanjing, China; diluted at 1:4000 in block solution) and Anti-Mouse lgG (purchased from Sigama, St. Louis, MO, USA; diluted at 1:5000 in block solution). Finally, the signal was developed with the Tanon 5200 fully automated luminous system (Shanghai, China).

### 2.5. Enzymatic Reaction In Vitro 

The enzyme activity was tested in vitro as described by Fraser [19]. Briefly, the enzyme reaction buffer (containing 0.4 M Tris-HCl pH 8.0, 1 mM dithiothreitol, 0.5 mM FeSO_4_, 5 mM ascorbic acid, 0.5 mM 2-oxoglutarate, and 0.1% *w*/*v* deoxycholate) was mixed with the protein crude extracts from *E. coli*, followed by an additional 2 mM NADPH and 2 mM ATP. The mixture was balanced for 5 min at 30 °C in a shaker with 250 rpm. Then, 100 μg β-carotene was added as the substrate, and the reaction was continued in the shaker with 250 rpm in the dark at 30 °C. After 3 h, the reaction was stopped, and the solution was stored in the refrigerator at −80 °C for further analysis. As the negative control, the enzymatic solution without protein crude extracts was also prepared.

### 2.6. Carotenoids Production in E. coli

For echinenone and canthaxanthin production from β-carotene, plasmids pG∆ZW-*CrtW_Bsp_*/HpW1/HpW2/CrW1/CzW1 coupled with or without pG-KJE8 were transformed into *E. coli* BL21(DE3), while plasmids p*CrtW_Bsp_*, pHpW1, pHpW2, pCrW1, and pCzW1 coupled with or without pG-KJE8 were transformed into *E. coli* BL21(DE3) harboring pACCAR16Δ. Bacterial colonies were picked from the LB selection plate and inoculated into 3 mL of LB medium containing proper antibiotics. After incubation overnight at 37 °C with shaking at 220 rpm, the culture was inoculated into 100mL of LB medium at the ratio of 1:100 and grown at 30 °C until OD600 reached 0.6. IPTG was then added to the culture at a final concentration of 0.1 mM or 0.5 mM, depending on the type of plasmids within *E. coli*. The culture was then maintained for an additional 6 h at 30 °C for carotenoids’ accumulation.

### 2.7. Preparation of Enzyme Reaction Solution 

The carotenoids were extracted from *E. coli* cells, as described previously [20]. To extract carotenoids from the enzymatic reaction mixture, 1/10 volume of 2 mol/L NaOH and 1/5 volume of 3 mol/L 10% SDS solution were added into the mixture, followed by vortexing for 30 s. Subsequently, 1/2 volume of 3 mol/L NaAc solution was added into the mixture, followed by another vortex for 30 s. The mixture was then centrifuged at 8000 rpm for 8 min at 4 °C, and the pellet was collected. Chloroform (1 mL) was used to dissolve carotenoids from the pellet. After centrifugation at 8000 rpm for 8 min at 4 °C, the supernatant containing carotenoids was collected, vacuum-dried, and redissolved with organic solution containing methanol and isopropanol (8:2, V:V).

### 2.8. HPLC Analysis

A high-performance liquid chromatograph system (LC-2030C 3D, Shimadzu, Kyoto, Japan) coupled with a chromatographic column (Phenomenex Inc., Aschffenburg, Germany, 150 × 3.0 mm, 5 μm) were used to detect and quantify β-carotene, echinenone, and canthaxanthin. For the analysis, the mobile phase A is ultrapure water, and the mobile phase B is a mixture with 80% methanol and 20% isopropanol. The flow rate is 0.6 mL/min, and the column temperature is 35 °C. The injection volume was 5 μL. The gradient elution conditions were as follows: 0 min 15% A and 85% B; 0–15 min 0% A and 100% B; 10–15 min 0% A and 100% B; 15.1–20 min 15% A and 85% B. The absorbance at 450 nm for 20 min was collected for analysis.

To evaluate the content of targets in the examined samples, the absorbance of serial dilutions of the canthaxanthin, echinenone, and β-carotene standard solutions (purchased from Sigma, Missouri, USA) were tested to create a calibration curve, using concentrations as the x-axis and their corresponding peak area as the y-axis.

### 2.9. Data Analysis and Statistics

All experiments were replicated in triplicate as biological repeats. Data were statistically analyzed using SPSS23.0, displayed with GraphPad7.0, and expressed as mean ± standard deviation.

## 3. Results

### 3.1. Soluble Production and Activity of β-Carotene Ketolase Using Different Vectors 

**Figure 2:**
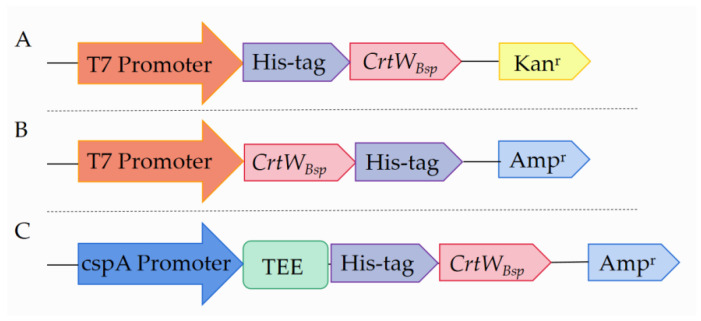
Anatomy of expression vectors. The figure depicts major features presented in three evaluated expression vectors in this study. (**A**) Anatomy of plasmid p30a*CrtW_Bsp_*; (**B**) anatomy of plasmid p32a*CrtW_Bsp_*; (**C**) anatomy of plasmid pCold-*CrtW_Bsp_*.

To investigate the effect of different expression vectors on β-carotene ketolase, plasmids p30*CrtW_Bsp_*, p32*CrtW_Bsp_* and pCold-*CrtW_Bsp_* were constructed and expressed in *E. coli* BL21 (DE3). In comparison, p30*CrtW_Bsp_* has the kanamycin selection marker and His-tag at the N-terminator of *CrtW_Bsp_*, while p32*CrtW_Bsp_* has the ampicillin selection marker and His-tag at the C-terminator of *CrtW_Bsp_* (Figure 2A,B). The *CrtW_Bsp_* transcripts were driven by T7 promoters in both p30*CrtW_Bsp_* and p32*CrtW_Bsp_*. Differently, the transcription of *CrtW_Bsp_* in pCold-*CrtW_Bsp_* was driven by the cspA promoter. Furthermore, the translation-enhancing element TEE was fused at the N-terminator of *CrtW_Bsp_*, in addition to His-tag (Figure 2C).

**Figure 3:**
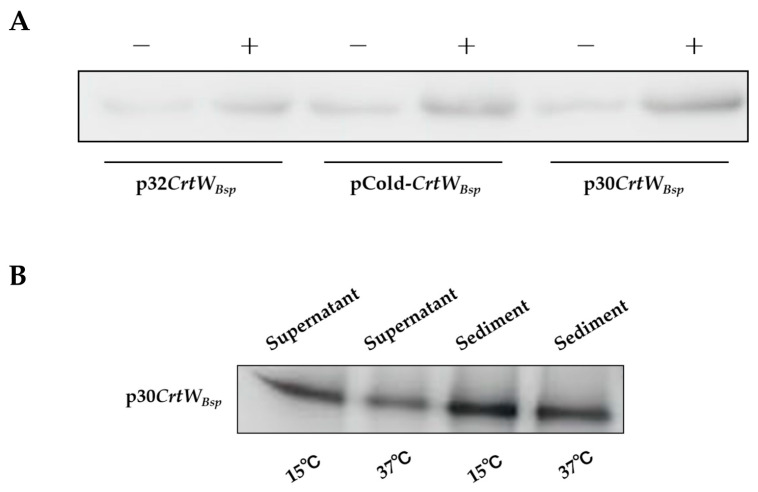
Comparison of the production of soluble proteins by *E. coli* cells harboring p30*CrtW_Bsp_*, p32*CrtW_Bsp_*, and pCold-*CrtW_Bsp_*. (**A**): Western blot analysis of soluble production of β-carotene ketolase protein by pET-32a, pCold, and pET-30a vectors. Proteins were extracted from *E. coli* cells through being induced (+) or not induced (−) with IPTG; (**B**): Western blot analysis of soluble production of β-carotene ketolase protein extracted from *E. coli* cells with p30*CrtW_Bsp_* cultured at different temperatures.

According to the Western blot result using protein crude extracts isolated form the supernatant of cell disrupts, a protein with molecular weight of 28.1 kD was presented, which is consistent with the theoretical expected value of the *CrtW_Bsp_* protein (Figure 3A), indicating the successful production of *CrtW_Bsp_* by all three plasmids in *E. coli*. Based on the intensity of the protein shown on the filter, the content of the target protein from *E. coli* harboring p30*CrtW_Bsp_* was about 3.8 times more than that from *E. coli* with p32*CrtW_Bsp_* (Figure 3A). In *E. coli* harboring pCold-*CrtW_Bsp_*, which has the cold shock promoter instead of the T7 promoter, the production of the target protein was increased by about 2.4 times, compared with that harboring p32*CrtW_Bsp_* (Figure 3B). Therefore, among three expression vectors, p30*CrtW_Bsp_* generated the most soluble proteins in *E. coil*. Furthermore, it was found that the amount of soluble protein was further accumulated when *E. coli* cells were cultured at 15 °C rather than at 37 °C, even though the protein was mainly in the insoluble form (Figure 3B).

**Figure 4:**
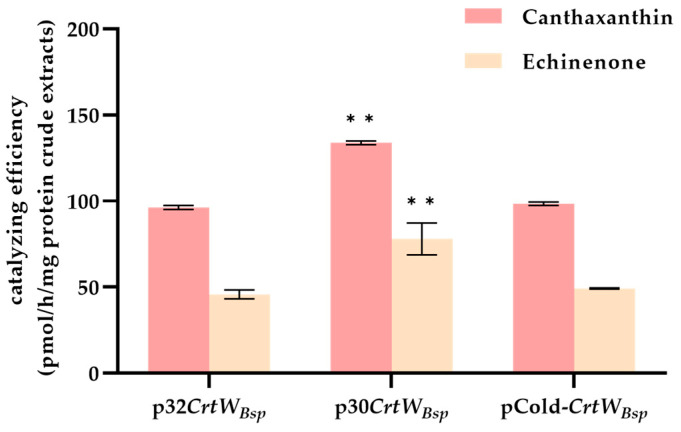
Comparison of in vitro enzyme activity of soluble proteins obtained from *E. coli* cells harboring p30*CrtW_Bsp_*, p32*CrtW_Bsp_*, and pCold-*CrtW_Bsp_*. The error bar represents the standard deviation from three independent experiments. ** indicates the significance between two means at the level of 0.01, respectively.

In order to further investigate if the activity of β-carotene ketolase was varied when produced in *E. coli* with p30*CrtW_Bsp_*, p32*CrtW_Bsp_*, and pCold-*CrtW_Bsp_*, in vitro enzyme activity was performed. Results showed that on the basis of having β-carotene as the substrate, β-carotene ketolase driven from p32*CrtW_Bsp_* and pCold-*CrtW_Bsp_* performed similarly in echinenone and canthaxanthin production. In contrast, β-carotene ketolase from p30*CrtW_Bsp_* was more efficient in the production of echinenone and canthaxanthin, at the rate of 133.8 pmol/h/mg and 78.0 pmol/h/mg, respectively (Figure 4). 

### 3.2. Effect of Molecular Chaperone on the Soluble Production and Activity of β-Carotene Ketolase

**Figure 5:**
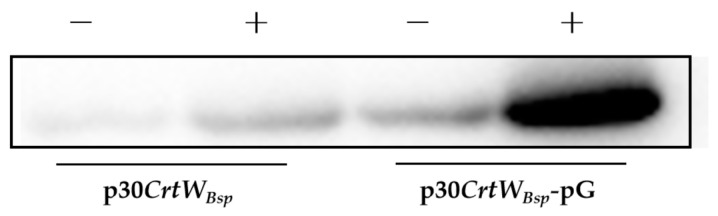
Western blot analysis of soluble production of β-carotene ketolase in *E. coli* cells harboring p30*CrtW_Bsp_* with (+) or without (−) the molecular chaperone pG-KJE8. Proteins were extracted from *E. coli* cells treated or untreated with IPTG and L-arabinose.

To investigate if the molecular chaperone can improve the content and activity of β-carotene ketolase in protein extracts, the commercial vector pG-KJE8 containing elements of GroEL, GroES, GrpE, DnaK, and DnaJ was employed and co-expressed with p30*CrtW_Bsp_* in *E. coli*. According to the Western blot analysis, the expected protein with a molecular weight of 28.1 kD was presented (Figure 5). Based on the gray intensity evaluation, the amount of soluble protein produced in *E. coli* with pG-KJE8 was 8.0 times that without pG-KJE8, indicating the excellent performance of the molecular chaperone in the enhancement of the solubility of β-carotene ketolase in *E. coli* (Figure 5). 

**Figure 6:**
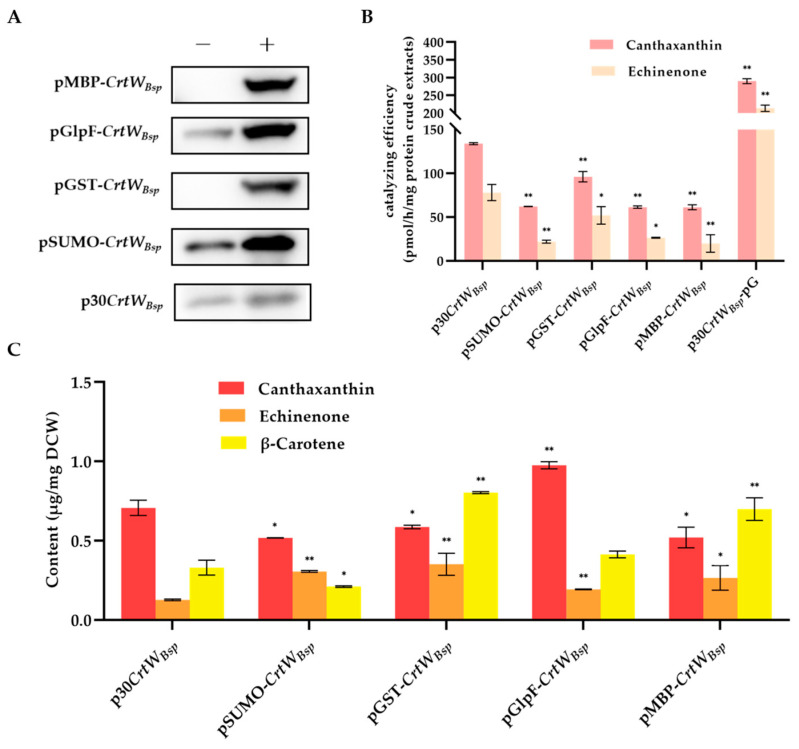
Effect of soluble fusion tags on the soluble production and activity of β-carotene ketolase. (**A**): Western blot analysis of soluble production of β-carotene ketolase protein with p30*CrtW_Bsp_*-pG, pSUMO-*CrtW_Bsp_*, pGST-*CrtW_Bsp_*, pGlpF-*CrtW_Bsp_* and pMBP-*CrtW_Bsp_*. Proteins were extracted from *E. coli* cells induced (+) or not induced (−) with IPTG; (**B**): comparison of in vitro enzyme activity of soluble proteins obtained from *E. coli* cells harboring p30*CrtW_Bsp_*, pSUMO-*CrtW_Bsp_*, pGST-*CrtW_Bsp_*, pGlpF-*CrtW_Bsp_*, pMBP-*CrtW_Bsp_* and p30*CrtW_Bsp_*-pG; (**C**): the effect of fusion tags on the in vivo activity of β-carotene ketolase. The error bar represents the standard deviation from three independent experiments. * and ** indicates the significance between two means at the level of 0.05 and 0.01, respectively.

Further in vitro enzyme activity assays revealed that with the help of pG-KJE8, the production of both canthaxanthin and echinenone was dramatically increased from 133.8/78.0 pmol/h/mg (canthaxanthin/echinenone) to 290.1/213.3 pmol/h/mg (canthaxanthin/echinenone), respectively (Figure 6B).

### 3.3. Effect of Fusion Tags on Soluble Production and Activity of β-Carotene Ketolase 

In order to further enhance the solubility of the protein since most target protein was presented in the sediment of *E. coli* cells (Figure 3B), four fusion tags including SUMO, GST, GlpF, and MBP were added to the N-terminus of *CrtW_Bsp_* in plasmid p30*CrtW_Bsp_* to generate fusion proteins. After induction with IPTG at 15 °C, protein extracts from *E. coli* cells with SUMO-*CrtW_Bsp_*, GST-*CrtW_Bsp_*, GlpF-*CrtW_Bsp,_* and MBP-*CrtW_Bsp_* were analyzed. Western blot showed that proteins with molecular weights of 40.5 kD, 50.3 kD, 57.7 kD, and 68.3 kD were presented as expected, indicating the successful production of fusion protein by all plasmids in *E. coli* (Figure 6A). Compared with the molecular chaperone, the soluble protein was significantly increased with the help of all fusion tags, by 3.3 times for GST, 2.7 times for SUMO, 3.6 times for MBP, and 3.2 times for GlpF, respectively (Figure 6A).

Further in vitro enzyme activity assays using target proteins containing fusion tags were performed to determine whether the activity of β-carotene ketolase was affected by the additional tags located at the N-terminator of the target protein. Results suggested their corresponding enzyme activities did not increase significantly as expected, since the fusion tags improved the solubility of β-carotene ketolase (Figure 6A). Conversely, their enzyme activities were significantly decreased compared with those driven from p30*CrtW_Bsp_*, evidenced by the decreased amount of echinenone and canthaxanthin detected in the enzymatic reaction solution (Figure 6B).

Furthermore, the in vivo activity of β-carotene ketolase with different fusion tags was also determined. A similar result was observed, so the production of canthaxanthin was decreased, with the exception of β-carotene ketolase with GlpF tag which improved the production of canthaxanthin from 0.71 μg/mg DCW to 0.98 μg/mg DCW (Figure 6C). In contrast, the production of echinenone was significantly increased with the help of all four fusion tags (Figure 6C). It is indicated that the impact of β-carotene ketolase on keto of the second group of β-carotene was inhibited by fusion tags. It is noted that the *E. coli* with pGlpF-*CrtW_Bsp_* produced the highest amount of canthaxanthin though the catalyzing efficiency of its β-carotene ketolase, which was lower than the control p30*CrtW_Bsp_* in vitro, implying that the GlpF tag was aid for the enzymatic activity of β-carotene ketolase in *E. coli* cells.

### 3.4. Effect of Molecular Chaperone on the Soluble Production and Activity of Other β-Carotene Ketolases Derived from Microalgae

**Figure 7:**
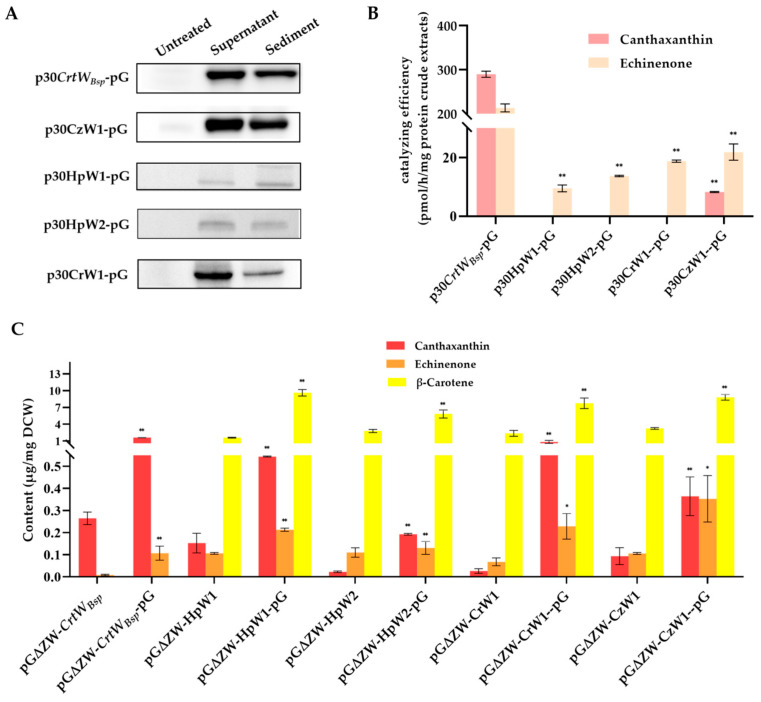
The effect of pG-KJE8 on the soluble production and activity of β-carotene ketolase from different organisms. (**A**) Western blot analysis of soluble production of β-carotene ketolase protein under the presence of pG-KJE8 in *E. coli.* Proteins were extracted from *E. coli* cells being induced (+) or not induced (−) with IPTG and L-arabinose; (**B**) the enzymatic activity of β-carotene ketolase isolated from *E. coli* cells harboring different plasmids; (**C**) the content of canthaxanthin, echinenone, and β-carotene in *E. coli* cells containing β-carotene synthesis gene cluster and different genes encoding β-carotene ketolase, with or without the additional pG-KJE8. The error bar represents the standard deviation from three independent experiments. * and ** indicates the significance between two means at the levels of 0.05 and 0.01.

To see if the molecular chaperone also works well on other β-carotene ketolases for the improvement of soluble-protein content, plasmids pHpW1, pHpW2, pCrW1, and pCzW1 were constructed and co-expressed with pG-KJE8 in *E. coli* BL21(DE3). According to the Western blot analysis, proteins with a molecular weight of 37.2 kD, 42.6 kD, 48.4 kD, and 36.2 kD were presented as predicted for HpW1, HpW2, CrW1, and CzW1, respectively, indicating the successful production of proteins by all plasmids in *E. coli* (Figure 7A). Moreover, according to the gray intensity, proteins of β-carotene ketolases *CrtW_Bsp_*, CzW1, HpW2, and CrW1 mainly existed in a soluble form in the supernatant. However, the production of β-carotene ketolase derived from different species of microalgae was dramatically varied in *E. coli*. It seemed like β-carotene ketolases encoded with HpW1 and HpW2 were accumulated much less than those with *CrtW_Bsp_*, CzW1, and CrW1 (Figure 7A). Consistently, the in vitro enzyme activity assays showed that protein extracts from *E. coli* harboring pHpW1 and pHpW2 generated less echinenone than others, which only generated 4.45% and 6.42% of that from harboring p30*CrtW_Bsp_* (Figure 7B). It is surprising that canthaxanthin was only detected in enzymatic solution containing protein extracts from *E. coli* with p*CrtW_Bsp_* and pCzW1 (Figure 7B). Moreover, although the level of soluble β-carotene ketolase in *E. coli* with pCzW1 was significantly higher than that with p*CrtW_Bsp_*, the content of canthaxanthin in the enzymatic solution was much higher in p*CrtW_Bsp_*-pG than in pCzW1-pG, revealing a higher in vitro substrate catalytic efficiency of β-carotene ketolases encoded with *CrtW_Bsp_* (Figure 7B). 

In contrast, the in vivo analysis revealed that β-carotene ketolase from HpW1, HpW2, and CrW1 can also generate canthaxanthin within *E. coli* cells when co-existing with a gene cluster synthesized with β-carotene (Figure 7C). Additionally, the content of canthaxanthin and echinenone in *E. coli* cells was significantly increased when the molecular chaperone was presented (Figure 7C). On the other hand, the β-carotene was not found in *E. coli* cells with p30*CrtW_Bsp_*, demonstrating its maximum catalyzing efficiency compared with other microalgae derived from β-carotene ketolase genes. By comparing the productivity, the effect of the molecular chaperone was varied among these β-carotene ketolase. The maximum increment of canthaxanthin production was found in pG∆ZW-CrW1-pG, which is 30.77 times that in pG∆ZW-CrW1, while the least increment was found in pG∆ZW-HpW1-pG, which is 2.57 times that in pG∆ZW-HpW1. 

## 4. Discussion

Currently, *E. coli* stands as one of the most advanced expression systems due to its clear genetic background, stable expression, and cost-effectiveness. However, this system is susceptible to interference from membrane proteins and toxic proteins, resulting in low or even no protein expression at all [16]. β-carotene ketolase is a key enzyme for the biosynthesis of economically important keto-form carotenoids, such as canthaxanthin and astaxanthin [21]. Unfortunately, according to previous reports, β-carotene ketolase belongs to the membrane proteins [22,23]. Compared with water-soluble proteins, membrane proteins are very stable, so they fold and unfold very slowly [24]. Moreover, due to the thermodynamic instability of polar groups, the secondary structure of membrane proteins is more difficult to destroy than that of water-soluble proteins [25]. The formation of an inclusion body caused by slow and incorrect protein folding significantly hindered the performance of β-carotene ketolase in *E. coli*. It has been reviewed that the key thing for the soluble expression of membrane proteins in general could be the lowering of protein production and the enhancement of protein folding [26]. Hence, in this study, four strategies were evaluated to enhance the soluble production and catalyzing activity of β-carotene ketolase in *E. coli*, aiming to improve the production of canthaxanthin for further astaxanthin production improvement. 

First of all, the promoter is the most important element in a vector to drive the expression of the target gene. An ideal promoter should possess three key characteristics: (1) it should facilitate a high expression of the target gene; (2) it should minimize background expression and prevent elements negatively impacting the host’s logarithmic growth phase; and (3) the induction method should be simple and cost-effective [27,28]. In this study, the commonly used T7 promoter and a cold shock cspA promoter were employed to evaluate their effect on protein accumulation (Figure 2). The recombinant protein *CrtW_Bsp_* showed a higher level of soluble-protein accumulation under the T7 promoter, compared to the cspA promoter (Figure 3B). It is interesting that more *CrtW_Bsp_* accumulated when using pET-30a as the carrier than when using pET-32a as the carrier, even though these two vectors share the same expression cassette and are mainly different in the selection marker, leading to the prediction that the type of antibiotic might also affect the accumulation of the target protein through affection on the proteome of *E. coli* cells. 

The optimum growth temperature of *E. coli* is 37 °C. However, a high temperature will accelerate protein synthesis in *E. coli* and lead to misfolding and the formation of inclusion bodies [27]. Therefore, protein aggregation is the bottleneck of *E. coli* production of recombinant proteins. It has been reported that the growth rate of bacteria can be slowed down under a low temperature, thereby decelerating the formation of aggregates of folding intermediates, which is beneficial for the correct folding of proteins [28]. An example was previously reported that the proportion of recombinant GFP proteins in whole protein extracts was decreased following a decrease in temperature [29]. Results from this study also have the same finding that the amount of soluble fusion protein was higher in *E. coli* cells grown at 15 °C than at 37 °C (Figure 3B). 

It was observed that most target proteins still remained in the form of inclusion bodies (Figure 3B). Therefore, two other strategies were applied in an attempt to further release the soluble β-carotene ketolase, including the soluble-protein tag fused with the target protein and molecular chaperone. A study has proved that the incorporation of an MBP tag at the N-terminus of proteins increased the soluble expression of recombinant proteins in numerous prokaryotes and eukaryotes [30]. Other than MBP, three more tags were tested in this study, including SUMO, GST, and GlpF. It was found that all additional fusion tags significantly increased the amount of β-carotene ketolase in the protein crude extracts (Figure 6A). Among them, the GlpF tag not only serves as a fusion tag, but also as a membrane localization tag, guiding the target protein to locate on the *E. coli* membrane to increase the target yield [31], which might explain the highest production of canthaxanthin in *E. coli* cells with GlpF-*CrtW_Bsp_* (Figure 6B). On the other hand, the in vitro enzyme activity analysis showed that the additional tags reduced the catalyzing efficiency of β-carotene ketolase, possibly because of a changed spatial structure of *CrtW_Bsp_* from fusion tags. The introduction of fusion tag elements by Christoph Köppl et al. had a positive effect on the titer, but they found that it had obvious side effects on the batch fermentation of hFGD-2 production feedstock [32]. Therefore, in order to prevent steric hindrance, it is necessary to further remove the fusion protein’s tag, which can also be linked through the junction sequence ASASNGASA [22,33,34]. According to reports, the molecular chaperone could bind to the misfolded protein to unfold it; subsequently, the target is released from the molecular chaperone and has another opportunity to be folded correctly to enhance the secretion of soluble proteins and maintain protein stability [35,36]. There are several molecular chaperones that have been discovered, such as Sec, GroEL/GroES, Dna, tig, Hsp, etc. [26]. GroES acts as an auxiliary molecule to GroEL to form a cage-like structure that binds to ATP and unfolded proteins in a cis conformation, gradually releasing GroES, ADP, and the protein from the trans conformation [37]. In the DnaK/DnaJ/GrpE system, DnaJ and GrpE act as auxiliary molecular chaperones to DnaK; DnaJ promotes the binding of DnaK to substrates by hydrolyzing ATP that is bound to DnaK; and GrpE catalyzes the conversion of ADP to ATP, facilitating the release of the substrate [38]. It was found that pG-KJE8 encodes the GroEL/GroES and DnaK/DnaJ/GrpE simultaneously and had the best performance on the soluble expression of *Psychrobacter* sp. lipase [26]. In another example, the active soluble form of ADH protein was obtained through co-expression with the chaperone protein using the pG-KJE8 vector [39]. By using the chaperone GroEs-GroEL as partners, the enzyme activity of ChSase AC II increased from 3.12 U/mL to 9.15 U/mL [40]. The production of astaxanthin was further increased to 26 mg/L and 6.17 mg/g DCW (dry cell weight) by utilizing the molecular chaperone genes groES-groEL [41]. Similar results were also observed in this study that the chaperone genes in pG-KJE8 significantly increased the canthaxanthin and echinenone production to as high as 30.77 times (Figure 6B and Figure 7B,C). In the future, the application of a molecular chaperone would be a good choice for the research of membrane protein when aiming to improve yield.

## 5. Conclusions

In this study, several attempts were made aiming to improve the soluble production and catalytic activity of β-carotene ketolase, which is a key enzyme involved in canthaxanthin and astaxanthin production and is hardly produced in *E. coli*. Results suggested that the production of soluble β-carotene ketolase in *E. coli* and its ability to convert β-carotene can be significantly increased with the application of a pET-30a vector, incubation at 15 °C, and co-expression with a molecular chaperone. Furthermore, a molecular chaperone also worked well with β-carotene ketolase from microalgae, particularly for soluble expression and enzymatic activity. This study provides a foundation for further research on the improvement of β-carotene ketolase activity, as well as new ideas for the improvement of carotenoid production.

## Data Availability

The authors promise that all data generated or analyzed in the present study are included in this article and in the additional information.

## Supplementary Materials

The following supporting information can be downloaded at https://www.mdpi.com/article/10.3390/microorganisms12020377/s1, Figure S1: HPLC diagram of standards and extracts from *E. coli* harboring pG∆ZW-CrW1-pG. Table S1: Plasmids constructed or adopted in this study. Table S2: Primers used in this study.
