# Supplementary material for: Improvement of Carotenoids’ Production by Increasing the Activity of Beta-Carotene Ketolase with Different Strategies
Source: Microorganisms. 2024 Feb 12;12(2):377. doi: 10.3390/microorganisms12020377 (PMC10891602; doi:10.3390/microorganisms12020377)
Supplement: Supplementary file 1 [file microorganisms-12-00377-s001.zip › microorganisms-2817310-supplementary.pdf]

Supplementary

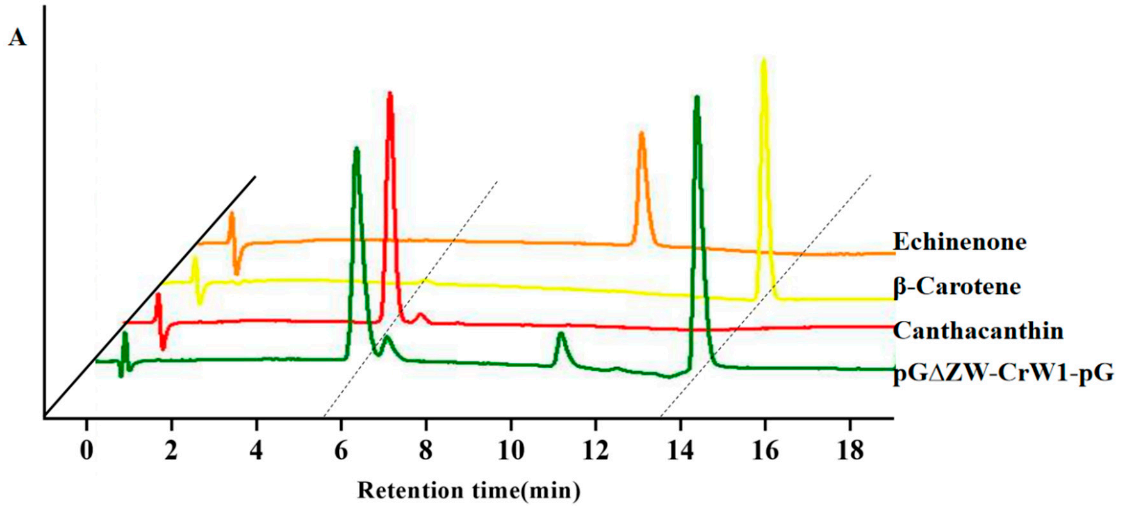

Figure S1. HPLC diagram of standards and extracts from *E.coli* harboring pGΔZW-CrW1-pG.

Table S1. Plasmids constructed or adopted in this study.

Plasmids	Plasmid information	Source
pACCAR16Δcrtx	pET, T7, HpE, CrtI, CrtB, CrtY, Chl^r	Shanghai Jiaotong University
pG-KJE8	pET, T7, Dnak, DnaJ, GrpE, GroES, GroE, Chl^r	General Biosystems (Anhui) Co., Ltd
p30$CrtW_{Bsp}$	pET, T7, $CrtW_{Bsp}$, Kan^r	This study
p32$CrtW_{Bsp}$	pET, T7, $CrtW_{Bsp}$, Amp^r	This lab
pCold-$CrtW_{Bsp}$	pET, cspA, $CrtW_{Bsp}$, Amp^r	This study
pGST-$CrtW_{Bsp}$	pET, T7, GST, $CrtW_{Bsp}$, Kan^r	This study
pMBP-$CrtW_{Bsp}$	pET, T7, MBP, $CrtW_{Bsp}$, Kan^r	This study
pGlpF-$CrtW_{Bsp}$	pET, T7, GlpF, $CrtW_{Bsp}$, Kan^r	This study
pSUMO-$CrtW_{Bsp}$	pET, T7, SUMO, $CrtW_{Bsp}$, Kan^r	This study
pHpW1	pET, T7, HpW1, Kan^r	This study
pHpW2	pET, T7, HpW2, Kan^r	This study
pCrW1	pET, T7, CrW1, Kan^r	This study
pCzW1	pET, T7, CzW1, Kan^r	This study
pGΔZW-$CrtW_{Bsp}$	pET, T7, HpE, CrtI, CrtB, CrtY, $CrtW_{Bsp}$, Amp^r	This lab
pGΔZW-HpW1	pET, T7, HpE, CrtI, CrtB, CrtY, HpW1, Amp^r	This lab
pGΔZW-HpW2	pET, T7, HpE, CrtI, CrtB, CrtY, HpW2, Amp^r	This lab
pGΔZW-CrW1	pET, T7, HpE, CrtI, CrtB, CrtY, CrW1, Amp^r	This lab
pGΔZW-CzW1	pET, T7, HpE, CrtI, CrtB, CrtY, CzW1, Amp^r	This lab

Table S2. Primers used in this study.

ID	Primer sequence（5′→3′）	used for
HpW1F	CAGTcatatgCACCACCACCACCACCACCAGCTGGCCGCCACCGTG	pHpW1 construction
HpW1R	ACTGaagcttCTTATGCCGGCACCAGAC	
HpW2F	CAGTcatatgCACCACCACCACCACCACCAGGTGCATGCAGGTCAG	pHpW2 construction
HpW2R	ACTGaagcttCTTATGCCAGGGCAGGCA	
CrW1F	CAGTcatatgCACCACCACCACCACCACGGTCCGGGTATTCAGCCG	pCrW1 construction
CrW1R	ACTGaagcttCTTAGGCCATAACGCCCA	
CzW1F	CAGTcatatgCACCACCACCACCACCACGCCCCTGATGTGACCCAT	pCzW1 construction
CzW1R	ACTGaagcttCTTAATTCACCAGCTGAG	

The restriction sites are indicated by lowercase letters.
